# A Brief History of Protein Sorting Prediction

**DOI:** 10.1007/s10930-019-09838-3

**Published:** 2019-05-22

**Authors:** Henrik Nielsen, Konstantinos D. Tsirigos, Søren Brunak, Gunnar von Heijne

**Affiliations:** 1 Department of Health Technology, Section for Bioinformatics, Technical University of Denmark, Kgs. Lyngby, Denmark; 2 Faculty of Health and Medical Sciences, Novo Nordisk Foundation Center for Protein Research, University of Copenhagen, Copenhagen, Denmark; 3 Department of Biochemistry and Biophysics, Stockholm University, Stockholm, Sweden; 4 Science for Life Laboratory, Stockholm University, Solna, Sweden

**Keywords:** Signal peptides, Protein sorting, Bioinformatics, Prediction

## Abstract

Ever since the signal hypothesis was proposed in 1971, the exact nature of signal peptides has been a focus point of research. The prediction of signal peptides and protein subcellular location from amino acid sequences has been an important problem in bioinformatics since the dawn of this research field, involving many statistical and machine learning technologies. In this review, we provide a historical account of how position-weight matrices, artificial neural networks, hidden Markov models, support vector machines and, lately, deep learning techniques have been used in the attempts to predict where proteins go. Because the secretory pathway was the first one to be studied both experimentally and through bioinformatics, our main focus is on the historical development of prediction methods for signal peptides that target proteins for secretion; prediction methods to identify targeting signals for other cellular compartments are treated in less detail.

## Introduction

The Signal Hypothesis was first proposed by Günter Blobel and David D. Sabatini in a short speculative paper in 1971 [1], where they wrote: “All mRNA’s to be translated on bound ribosomes are assumed to have a common feature such as several codons near their 5′ end, not present in mRNA’s which are to be translated on free ribosomes. The resulting common sequence of amino acids near the N-terminal of the nascent chains or a modification of it would then be recognized by a factor mediating the binding to the membrane.” The postulated “common feature” was first seen in 1972 by Milstein et al. as a larger precursor form of immunoglobulin light chains synthesized in vitro in the absence of rough microsomes [2]. The definitive proof for a cleavable signal peptide (SP) directing protein translocation into the lumen of the ER was published in 1975 by Günter Blobel and Bernhard Dobberstein in their two classic papers describing the in vitro reconstitution of protein translocation [3, 4].

The next obvious question was: what do SPs look like? Are they highly conserved, as suggested by Blobel and Sabatini, or perhaps of more variable sequence? The first data came in 1975 from Edman degradation of an immunoglobulin light chain precursor that had been radiolabeled by [^3^H]-Leu [5]. The data indicated that the light chain was synthesized with a 20-residue N-terminal extension containing Leu residues in positions 6–8 and 11–13, implying that the SP had a rather hydrophobic character. This was borne out when the full sequences of SPs were starting to be obtained by cDNA sequencing. The first statistical analyses were published in 1979 [6, 7]; they were based, respectively, on collections of 9 and 21, mainly eukaryotic, SPs and noted a semi-conserved positively charged N-terminus, a hydrophobic segment, and a C-terminal segment predicted to form a β-strand structure. The 21-sequence collection was analyzed by Garnier et al. in 1980 [8], with similar conclusions.

Sequence patterns around the signal peptidase cleavage site were first discussed in two papers published in early 1983, based on collections of 30 [9] and 78 [10] SPs, respectively; both papers reported that the cleavage site is characterized by having residues with small, uncharged side chains in positions − 1 and − 3 relative to the cleavage site, an observation referred to as the (− 3, − 1)-rule.

The first prediction method for SP cleavage sites was described in the paper that introduced the (− 3, − 1)-rule in 1983 [10]. It was based on a reduced-alphabet weight matrix combined with a rule for narrowing the search region. The weight matrix covered positions − 5 to + 1 relative to the cleavage site, using only seven different weights at each position, corresponding to groups of amino acids (AAs) with similar characteristics. The weight values were estimated manually rather than calculated from the data. The weight matrix score was only calculated for positions 12–20 counted from the beginning of the h-region—defined as the first quadruplet of AAs with at least three hydrophobic residues—and the cleavage site was assigned to the position with the highest score. This procedure could place the cleavage site correctly in 92% of the data used to estimate it, but measured on a larger data set a few years later, the test performance was only 64% [11]. This serves as a reminder that even a simple method such as a weight matrix can be overfitted, if the underlying data foundation is sparse.

However, this was not the first protein sorting prediction paper. A contender for that title would be the short article by Capaldi and Vanderkooi from 1972 [12], where they show that the proportion of polar residues is different between soluble proteins and integral (at the time called “intrinsic”) membrane proteins. At a cutoff of 40% polar residues, roughly half of the membrane proteins were identified with a false positive rate of 6%. This modest success rate was improved by Barrantes, who in papers from 1973 and 1975 [13, 14] developed a linear discriminant function based on two variables: the ratio between charged and hydrophobic AAs, and the average hydrophobicity according to Tanford [15]. While these early works described classification of entire proteins based on overall AA composition, the recognition of individual transmembrane (TM) helices based on AA sequence was pioneered by Kyte and Doolittle [16].

In this review, we describe the early history of protein sorting prediction in detail, while later developments will be mentioned only briefly. The purpose is not to make a complete list of protein sorting prediction software, but to describe those methods that imply significant developments in methodology—one could term them algorithmic paradigm shifts. The main focus will be on SP prediction, with additional sections on TM protein prediction and multi-category protein subcellular location prediction. Specific prediction methods for other sorting signals such as transit peptides for mitochondria and chloroplasts [17, 18], nuclear localization signals [19, 20] and peroxisomal targeting signals [21, 22] will not be mentioned, and are discussed in other, more general, reviews [23–25].

## Signal Peptide Prediction

Prediction of SPs involves two sub-tasks: discriminating between SPs and non-secretory proteins, and predicting the position of the SP cleavage site. It is important to keep in mind that the presence or absence of an SP is not equal to the question of whether the protein is secreted or not. On the one hand, proteins with SPs may be retained in the membrane or in one of the compartments of the eukaryotic secretory pathway (endoplasmic reticulum, Golgi apparatus or lysosomes); on the other hand, certain proteins are secreted without SPs, especially in bacteria [26], but also in eukaryotes [27].

### A Feature-Based Method

After the reduced weight matrix method appeared in 1983, the first SP predictor was published in 1985 by McGeoch [28] who tested a number of different sequence-derived features to find a combination providing good discrimination between SPs and other sequences. Identification of the precise cleavage site location was not attempted. The two selected features were: length of the uncharged region, and maximal hydrophobicity (on the scale of Kyte and Doolittle [16]) in an 8-AA window. The uncharged region was defined to begin after the last charged AA among the first 11 positions and to end at the next charged residue, while the maximal hydrophobicity was calculated 18 positions downstream from the start of the uncharged region. A non-linear discriminative function, separating the positive and negative examples in the plane defined by these two features, was determined manually.

Originally, this method was based on a very limited data set focusing primarily on virus proteins and immune system proteins, and it could not automatically be transferred to another training set because of the subjective element involved in drawing the separating curve through the two-dimensional feature space. However, the method was later integrated into the multi-category subcellular location predictor PSORT (see Sect. 4), where it is used in combination with von Heijne’s 1986 weight matrix (see Sect. 2.2). In PSORT I, the original two features were used for eukaryotic data [29], but for prokaryotic data, the method was retrained using discriminant analysis, and a third feature (net charge of the charged region) was incorporated [30]. For the newer PSORT II, the method has been further refined for yeast and *Bacillus subtilis*, optimizing not only the coefficients for the features in the discriminant function but also the parameters used to derive the features, i.e., the length of sequence regions scanned for charged or hydrophobic residues, and the hydrophobicity scale [31].

This example nicely illustrates the strengths and weaknesses of rule- and feature-based methods. On the one hand, it is quite transparent how each individual prediction was reached, based on features that are easy to calculate; but on the other hand, the generalization ability is limited. As an example, the rule for finding the start of the “uncharged region” imposes a hard limit on the length of the n-region, so that if an SP has a long n-region containing a charged residue after position 11 (there was one such example in the original data set), the “uncharged region” will not contain the h-region, but only a short arbitrary stretch from the n-region. The feature(s) derived from this will probably be totally out of range for SPs, leaving the method no chance of producing a reasonable answer.

### Weight Matrix Methods

A “real” position-weight matrix, calculated with log-odds scores, was published by von Heijne a few years later [11]. A range of window sizes was tested: Initially, positions − 15 to + 5 were used, but this could be narrowed to − 13 to + 2 without loss in performance. Separate matrices were calculated for prokaryotes and eukaryotes.

A common problem for position-weight matrices is that sometimes a certain AA is never observed at a certain position, making it impossible to calculate the logarithm needed. Actually, this situation is only the most extreme instance of the wider problem of sampling errors: The AA distributions are estimated from a limited number of examples, and this tends to overestimate the deviation from a random distribution. The solution is regularization: counteracting the sampling noise by modifying the distribution towards the background. In practice, this is done by adding *pseudocounts* to the observations before calculating the weights [32]. The regularization in this case was done in a rather ad hoc manner: No pseudocounts were added to non-zero counts, while counts of zero were set to one before log-transformation, except in positions − 1 and − 3 where counts of zero were considered to be significant and were set to 1/N (where N is the number of sequences).

When using the weight matrix for prediction, the weight matrix score was calculated for the first 40 positions of the protein chain, and the cleavage site was assigned to the position with the highest score. Thus, it is an example of a “moving window” method. The maximal weight matrix score in this region was also used for discrimination between SPs and other sequences.

This weight matrix has found extremely wide usage. It was never presented as a mail-server or web-server, but it has been made available as a downloadable program several times [33, 34], it is included in PSORT (see Sect. 4), and it is used together with the McGeoch method [28] in the commercial tool SPScan, which is a part of the widely used Wisconsin Package™ (Genetics Computer Group, GCG). It is also implemented as the “sigcleave” function in the public domain EMBOSS package [35].

In 2004, Hiller et al. made a new set of weight matrices named PrediSi [36] (separate for Gram-negative bacteria, Gram-positive bacteria and eukaryotes). There is no mention of regularization in the article, so apparently the authors did not observe any zero counts. Performance was reported to be close to that of SignalP 2.0 (see Sect. 2.4), but the PrediSi performance was measured by self-consistency only, i.e. without separate training and test sets.

In 2001 [37], Kuo-Chen Chou developed a simple method very similar to a weight matrix, although it was formulated in a different way. Instead of calculating log-odds and summing them over the window, he calculated probabilities and multiplied them (essentially, this is a zero order Markov chain). The probabilities were calculated separately for positive (cleavage site) windows and negative (non-cleavage site) windows and then subtracted to give a discrimination score. The performance was reported to be better than SignalP 1.0 [38] on the same data set, but the comparison was not valid, since it was not the same performance measure that was used. SignalP had reported that the proportion of SP *sequences* where the cleavage site had been correctly placed varied between 68% and 86% (depending on organism group), while Chou reported a 90% correct classification (on all organisms together) of cleavage site versus non-cleavage site *windows*. Essentially, this means that on average every tenth position in a random sequence would be marked as a cleavage site, and there was no indication of how often this result would allow the correct cleavage site to be identified.

Later the same year, Chou modified the method to include the so-called subsite coupling [39], meaning that correlations between selected positions were taken into account. Specifically, the conditional probabilities between positions − 3 and − 1 and between positions − 1 and + 1 were included in the calculations. The choice of exactly these positions rested on the fact that they differ most from the background composition, but this in itself is no indication that they are correlated. The modification gave a performance gain of a couple of percentage points. Still the same year, Chou published a version where correlations between all pairs of neighbour positions were used [40] (i.e., a first order Markov chain), which again resulted in a couple of percent better performance. Also in these two papers, the results are compared to the cleavage site performance of SignalP without acknowledging that the two kinds of percentages represent different performance measures.

The Signal-CF method from 2007 [41] is a further development of the method from Chou’s 2001 papers. It is a two-layer system which first determines whether a sequence is an SP or not and subsequently predicts cleavage sites if the sequence is predicted to be an SP. The first layer is based on the so-called pseudo AA composition [42], which essentially means the AA composition augmented by a number of autocorrelation terms that capture some of the sequence order effect by multiplying selected physicochemical parameters of AA pairs separated by a range of different distances. This is used as input to a modified version of the *k* nearest neighbours classifier. The second layer is a weight matrix with “subsite coupling” between positions − 3, − 1 and + 1 as described above. Performance was reported to be better than PrediSi, but was not compared to other predictors.

### Early Neural Network Methods

Artificial neural network algorithms (ANNs) learn from data iteratively presented to them by gradually adjusting their weights such that the output values eventually approach desired target values, for example 0.0 or 1.0 representing a dichotomy of whether a protein is secreted or not. Initially the ANNs used in the bioinformatics domain were linear perceptrons without hidden units, such as the ones used by Stormo et al. in 1982 to predict *E. coli* translational initiation sites in nucleotide sequences [43]. While such methods in some cases will outperform rule-based systems, the ANN methodology gained popularity following the reintroduction of the backpropagation algorithm in 1985 by the PDP group [44]. This algorithm allows for training of powerful non-linear models with hidden units that can change their output values significantly in response to small variations in input, for example a change of a single amino acid in a window into the sequence. The backpropagation algorithm was discovered numerous times, however, the pedagogical presentation by Rumelhart et al. [45] quickly led to it becoming widely used, much like the deep learning revolution of today [46]. Non-linear, feed-forward ANNs are quite agile as it is relatively easy to limit overfitting by reducing the number of hidden units [47]. Another feature that has added to the flexibility of the method is that it often is advantageous to combine networks, either in cascades or in a single step [48], a principle allowing for combination of widely different complementary features. This aspect has also had strong impact on the success of ANNs in the protein sorting domain [38].

The first ANN for discrimination between SPs and cytoplasmic proteins was made by Ladunga et al. [49]. Cleavage site prediction was not attempted, and a moving window was not used; instead, the N-terminal part (set to 20 residues after initial testing) of each sequence was used as input. The network was trained with the tiling algorithm, a procedure which builds up the network topology during training, adding as many hidden neurons as necessary to classify all training data correctly [50]. Classifying all training data correctly may sound remarkable, but it leads to virtually guaranteed overfitting—by adding parameters that fit each data point exactly, the network becomes unable to see the forest for the trees. This was reflected in a rather poor test performance when the network was applied to data that had not been part of the training process.

Also in 1991, Arrigo et al. [51] reported that an unsupervised Kohonen network unexpectedly identified the SP region from a small set of human insulin receptor gene data. The Kohonen network, also called a self-organizing feature map, is an example of an unsupervised ANN, where “training” takes place without target values in the training set [50]. The Kohonen network has an input layer and a layer of computational units—the Kohonen nodes. The two layers are fully connected, so that each Kohonen node has a weight vector. The Kohonen nodes are arranged in a way that defines a topological neighbourhood for each node, e.g. a square lattice. When a training example is shown to the network, the Kohonen node whose weight vector is nearest to the input vector is selected. The weight vectors of the selected node and its neighbours within a certain radius are updated, so that they move closer to the input vector by a factor determined by a learning rate. The radius and learning rate decrease during training. In this way, the Kohonen nodes arrange themselves into a pattern that reflects the structure of the input data.

Arrigo et al. trained a network with 30 Kohonen nodes on non-overlapping windows from the cDNA of four human insulin receptor genes. In each sequence, one of the input patterns was extracted as singular in some not very clearly described way; and it turned out that the extracted pattern was wholly or partly within the DNA coding for the SP for a wide range of window sizes. However, it is not clear whether this result has anything to do with SPs at all. Since the approach was not tested on proteins without SPs, the only conclusion to be drawn from this is that the initial part of the reading frame of insulin receptors is in some way peculiar. This might be due to the SP, but it might as well be the effect of correlation between codon bias and intragenic position [52].

Another early ANN was made by Schneider and Wrede [53, 54] who trained a feed-forward ANN to predict SP cleavage sites using moving windows. Instead of sparse encoding, seven physico-chemical properties were used to represent the sequence of AAs. After training networks with a single property at a time, four of them were selected to represent AAs in the final architecture. The training was done with a genetic algorithm rather than backpropagation. The computations were performed on an extremely small data set derived from *E. coli*: 17 sequences for training and 7 for testing. The final predictor had only 3 of the 7 test cleavage sites correctly placed when assigned by the highest score [54].

After training the predictor, it was used in a “simulated molecular evolution” experiment: a population of 12-aa sequence fragments were subjected to random changes and then selected based on their score for being putative signal sequence cleavage sites according to the ANN. After repeating this for many generations, a number of “optimal” cleavage sites were found, the precise sequence depending on the distance metric used [54]. Remarkably, these all contained Trp, especially at positions − 2 and − 5, and they had h-regions dominated by Phe. The highest-scoring cleavage site region was subsequently tested in vivo for their ability to promote secretion in an *E. coli* expression system [55]. Indeed, the Phe- and Trp-rich construct (FFFFGWYGWA↓RE) was fully cleavable, but so were the wild type (LAGFATVAQA↓AC) and a “consensus” pattern derived from a simpler, weight matrix-like approach (VVIMSASAMA↓AC).

Although this whole process is based on statistics from only 24 sequences, the result raises an interesting point: When using a linear method, the optimal example looks like a consensus of the training examples; but for a non-linear method, this is not necessarily the case. It is remarkable that the highest-scoring examples according to the ANN are very rich in otherwise rare AAs. So, is there any reason to expect that the non-linearly optimized “FFFFGWYGWA↓RE” is a more efficient cleavage site than the linearly optimized “VVIMSASAMA↓AC”? Probably not. Even if we assume that the peculiar residues are not just an effect of sampling error, the highest ANN score is found in a region of sequence space not covered by the training data, implying that the network score here is an *extrapolation* rather than an *interpolation*. And since ANNs do not contain any physicochemical model of how scores should vary with the input, but simply fit a non-linear function to the examples, a good generalization in interpolation does not necessarily mean a good generalization in extrapolation. The more non-linear the fitted function is, the less we can assume about how it should continue outside the region of the fitted data.

The Schneider and Wrede 1994 paper [54] was harshly criticized in a comment by Darius and Rojas [56] who, among other points, wrote: “The term “quality” for the value of the fitted function gives the impression that some biological significance is associated with values of the fitted function strictly between 0 and 1, but there is no justification for this kind of interpretation and finding the point where the fit achieves its maximum does not make sense.”

### SignalP and Related Neural Network Applications

SignalP 1.0 [38, 57] was in 1996 the first machine learning based SP prediction method to be available online as a web-server. SignalP used a combination of two different ANNs with moving windows: one trained to recognize all positions within the SPs, and one trained to recognize the cleavage site specifically. The outputs of these two networks were termed S-score and C-score, respectively. These were then combined into the Y-score, which was a function of the C-score and the slope of the S-score, used for predicting the location of the cleavage site. This way of combining two ANNs was inspired by the intron splice site predictor NetGene from 1991 [48].

SignalP 2.0 from 1999 [58] added a hidden Markov model (HMM, see Sect. 2.5) which made it possible to distinguish cleaved SPs from uncleaved signal anchors, while SignalP 3.0 from 2004 [59] introduced the D-score (the average of maximal Y-score and mean S-score) as a better discriminator between SPs and other sequences. SignalP 4.0 from 2011 [60] brought a new definition of the negative data: instead of just soluble intracellular proteins and signal anchors, it now included all TM proteins that had a TM helix within the first 70 positions and therefore could be mistaken for SPs. This drastically reduced the number of false positives produced by TM proteins. Unfortunately, SignalP 4.0 also had a lower sensitivity than SignalP 3.0 which led to many complaints from users whose favourite SPs were suddenly no longer positively predicted. Therefore, SignalP was in 2012 updated to version 4.1 with an option to choose an alternative threshold that reproduced the sensitivity of SignalP 3.0 [61].

In prokaryotes, there are several types of SPs. SignalP versions 1–4 were only able to predict the “standard” type of SP, which is transported through the Sec translocon and cleaved by signal peptidase I (also known as leader peptidase). However, there are also specialized SPs of prokaryotic lipoproteins which are cleaved by signal peptidase II (also known as lipoprotein signal peptidase); these have a different cleavage site motif with a 100% conserved cysteine in the +1 position [62]. In addition, there are SPs that direct their proteins through the Tat translocon; these have a characteristic twin-Arginine motif in the n-region [63] and are typically longer and less hydrophobic than Sec SPs [64]. In our group, separate ANNs were trained to predict such SPs, constituting the cores of the prediction methods LipoP from 2003 [65] and TatP from 2005 [66], respectively.

In 2003, an Italian group published SPEPlip [67], an ANN-based method very similar in architecture to SignalP. It was combined with a simple PROSITE pattern [68] which made it possible to distinguish between “standard” SPs cleaved by signal peptidase I and lipoprotein SPs cleaved by signal peptidase II.

The ANNs mentioned so far have generally had at most one hidden layer of computational neurons. The original backpropagation algorithm did not work well for deep networks with many layers, as the error made at the final output layer is not easy to use as a precise measure further up in the layered structure for adjusting the weights. In a deep ANN the many layers can filter and reorder features in very powerful ways. That is generally not possible using a single hidden layer unless one uses a huge number of units that most often will lead to overfitting. Deep ANN architectures are capable of carrying out sophisticated feature engineering as opposed to just establishing a simple decision boundary in the feature space resulting from the more moderate feature engineering achievable by the input-to-hidden transformation (when the hidden layer is of moderate size). The newer deep learning techniques solve these problems while keeping the number of adjustable parameters down. The techniques can be applied to feed-forward networks with many layers, but also to recurrent networks with loops that can memorize features that correlate with a desired output [69–72].

Deep learning in SP prediction was introduced in 2017 by the method DeepSig [73] (from the same group that published SPEPlip). It is based on convolutional ANNs, which can be described as a set of moving windows that look at small portions of the input sequence at a time. In DeepSig, there are three consecutive combinations of a convolutional layer feeding into an average pooling layer. This is followed by a so-called Taylor decomposition, a layer that estimates the relevance of each position in the input sequence for the classification of the sequence as an SP or not. Finally, the cleavage site is assigned by a grammar-restrained conditional random field, a probabilistic model which resembles an HMM in having a grammatical structure defining, in this case, the three regions of the SP. DeepSig was trained on the data from SignalP 4.0 and was reported to outperform it in most cases.

The recently released SignalP 5.0 [74] is based on deep ANNs of the recurrent type, where information flows not only from input to output, but also between the hidden units. The recurrent architecture of SignalP 5.0 makes it possible to abandon the moving windows, which defined the C- and S-scores in earlier versions of SignalP. Instead, the so-called long short-term memory networks can take a sequence of varying length as input and, if necessary, remember features from the beginning of the sequence while classifying positions further downstream [75]. The output from the long short-term memory layer is passed on to a conditional random field specifying that a cleavage site can only follow after an SP position and must necessarily be followed by a mature protein position. In this way, post-processing in the form of calculating Y- and D-scores becomes unnecessary.

Another innovation in SignalP 5.0 is that it now can predict SPs using the Tat pathway and lipoprotein SPs cleaved by signal peptidase II, meaning that a user no longer has to consult three different predictors in order to get a prediction of which type a prokaryotic SP belongs to.

### Hidden Markov Models

In SignalP 2 and 3 [58, 59], an HMM predicted SPs independently of the ANN. This HMM was not of the profile type that has found wide usage in databases of protein families such as Pfam [76]; instead, it reflected the usual description of SPs as consisting of n-, h-, and c-regions. The n-region and the h-region were modeled by common AA distributions; only around the cleavage site were single positions modeled separately. Instead of C-scores and S-scores, the HMMs provided probabilities of the three regions and of the cleavage site.

The original rationale for employing an HMM in SignalP 2 was to facilitate discrimination between SPs and signal anchors (uncleaved transmembrane helices close to the N-terminus). The distinction between SPs and signal anchors is not merely a question of having a cleavage site or not; signal anchors typically have hydrophobic regions longer than those of SPs. Interestingly, experiments have shown that it is possible to convert a cleavable SP to a signal anchor merely by lengthening the h-region [77, 78]. Our idea was that an HMM better than an ANN would be able to model this length difference. However, when constructing SignalP 4 [60] and retraining the HMMs on the new data set, we found them to be inferior in performance to the ANNs, thereby disproving our original idea. Apparently, ANNs with sufficiently large input windows are able to discriminate between short and long hydrophobic regions.

It is not impossible to construct a profile HMM that recognizes SPs; this was done by Zhang & Wood in 2003 [79]. However, its performance did not quite match that of the HMM module in SignalP 2.0.

The HMM-based Phobius TM topology prediction method from 2004 also includes an SP model [80]. This carries two advantages for TM prediction: first, false positive predictions of TM helices in SP regions are avoided; second, the topology of TM proteins carrying SPs is constrained by the fact that the N-terminus of the mature protein must be on the non-cytoplasmic side. The SP model in Phobius very closely resembles the one used in SignalP 2 and 3.

Similar to TatP [66] and LipoP [65], specialized prediction methods based on HMMs have also been presented. PRED-TAT [81] aims at discriminating between Tat and Sec-translocated SPs, as well as predicting their cleavage sites. PRED-LIPO [82] predicts presence of Sec/SPI SPs and Sec/SPII SPs in Gram-positive bacteria and can discriminate them from cytoplasmic and N-terminal TM proteins. Finally, PRED-SIGNAL [83] was the first computational method that specifically predicts SPs of archaeal origin and their cleavage sites, using an HMM approach.

### Support Vector Machine Applications

Unlike ANNs and HMMs, the third major machine learning algorithm, support vector machines (SVMs), has not played a big role in SP prediction. This is in contrast to the situation in prediction of subcellular location based on AA composition, where SVMs have been very important (see Sect. 4.1). One exception was made by Vert in 2002 [84], who trained an SVM for SP cleavage sites using a new class of kernels for strings. He used a − 8 to + 2 window and discriminated between windows with and without a cleavage site, and showed that the SVM was superior to a retrained weight matrix on the same data set. However, a comparison to an ANN was not made.

The year after, Cai et al. [85] published an SVM for predicting SP cleavage sites using sparse encoding of the inputs and a polynomial kernel. The resulting performance was not compared to anything, but it was slightly worse than the “subsite coupling” method by Chou [39] on the same dataset. In 2005, Wang et al. [86] tackled the same problem with a string kernel. They did an extensive comparison to a retrained weight matrix using the same dataset. For small windows (− 8 to + 2), the SVM outperformed the weight matrix, but for larger windows (− 13 to + 2 or larger) the advantage of the SVM disappeared.

Another SVM method is the TM topology predictor MEMSAT-SVM [87], which also predicts SPs. MEMSAT-SVM is built from five binary window-based classifiers, of which one is SP/non-SP. They are trained using the traditional polynomial or radial basis function kernels rather than a string kernel. MEMSAT-SVM is especially interesting because it can be compared to the ANN-based MEMSAT3 [88] which was published 2 years earlier. MEMSAT-SVM performed better than MEMSAT3 on almost all parameters.

Cleavage sites are not explicitly recognized by the windows of the MEMSAT methods, and the cleavage site performance is not reported in the papers. When testing version 4.0 of SignalP [60], we benchmarked MEMSAT3 and MEMSAT-SVM and found that both of them had cleavage site precision and recall values close to zero. Regarding discrimination between SPs and non-SPs, we could confirm that MEMSAT-SVM was better than MEMSAT3, but it was still not among the best performing methods.

### Homology-Based Methods

Signal-3L from 2007 [89] is a further development of the two-layer Signal-CF method [41] (see Sect. 2.2). It is mentioned in this section because it adds a third layer where alignment is used for improving the cleavage site prediction. The second layer suggests a number of cleavage sites, and then global pairwise alignment to a database of known SPs is used for selecting the best candidate among them. Performance was reported to be better than PrediSi [36], but was not compared to other predictors.

Signal-BLAST from 2008 [90] is a much simpler prediction method that runs BLAST [91] against a pre-constructed reference database of SPs, and, if it finds a hit with high similarity, it assigns the cleavage site position based on the homologous protein. This approach works very well if there are annotated close homologues in the database, but the drawback of this approach is that its performance solely depends on sequence similarities that can be detected by the BLASTP algorithm. The authors do not report on the tool’s performance when low or no sequence similarities are found. In our hands [74], Signal-BLAST did not perform well when no hits to its reference database were found, since it does not have a fallback strategy for these cases.

In 2017, Signal-3L was updated to version 2.0 [92] with a major modification of the architecture of the method. The first layer is now an SVM taking its input from PSI-BLAST [91] profiles, predicted secondary structure, predicted disorder, and selected physicochemical parameters, while the second layer searches the Conserved Domain Database [93] for functional domains in order to distinguish between SPs and TM helices. The third layer then corresponds to the second *and* third layer of the original Signal-3L. The performance was in some cases reported to be better than that of SignalP 4.1 [60], although SignalP 4.1 always had the lowest false positive rates. In the SignalP 5.0 benchmarks [74], however, Signal-3L 2.0 was not better than SignalP 4.1.

When using homology to predict SPs and their cleavage sites, it should be noted that SPs (and other N-terminal sorting signals) are actually *less* conserved than the mature regions of the proteins [94]. Therefore, it may be beneficial to search a database of entire proteins instead of a database of SPs.

## Transmembrane Protein Prediction

TM proteins constitute one of the most well-studied categories of membrane proteins. In numbers, they make up roughly 30% of the total number of proteins in a fully-sequenced organism, and their roles are diverse and important to the life of the cells [95, 96]. An important obstacle in the study of TM proteins is the difficulty in the determination of their 3D-structure, owing mainly to their hydrophobic nature [97]. The emergence of automated, computational methods that provide the researchers with a topological model of TM proteins has been very important to the field. These models inform about the number and position of the TM segments, alongside with the orientation with regards to the membrane. A major challenge in obtaining successful topology predictions is the exact same hydrophobic nature, which results in erroneous assignment of N-terminal TM segments as SPs and vice versa [98, 99].

As mentioned, Kyte and Doolittle in 1982 initiated the prediction of TM helices in a paper that was concerned with displaying the hydropathic character of proteins in general [16]. To this end, they developed a novel hydropathy index based on water/vapour transfer energies, buried/exposed propensities, and certain manual adjustments, the latter being described as “the result of personal bias and heated discussion between the authors”. Their program, SOAP, calculated the average hydropathy value of overlapping *k*-residue segments. While they found *k *= 9 to give the best correlation with buried and exposed stretches of globular proteins, *k *= 19 yielded the best discrimination between TM segments and hydrophobic stretches of globular proteins.

Later the same year, Argos et al. [100] published a method for predicting the structure of TM proteins. Instead of settling for one hydrophobicity scale, they investigated nine different properties of AAs and used a fitting procedure to the proposed structure of bacteriorhodopsin to adjust the weights for each property. Five of the nine properties were eventually selected. Instead of calculating averages within a fixed length window, a smoothing procedure was used. While a good agreement with the bacteriorhodopsin structure was achieved, the method was not very good at discriminating between TM segments and globular proteins.

The ALOM method from 1985 [101] was very similar to SOAP, but based on a larger data set. Four different hydrophobicity scales were tested, and the Kyte-Doolittle hydropathy was eventually chosen. The authors found a 17-residue window to give the best discrimination between integral and peripheral membrane proteins, and devised an additional procedure for assigning the precise boundaries between TM helices and loops. ALOM was later incorporated as a feature in the PSORT prediction method (see Sect. 4).

These early topology prediction methods were based on the amino acids’ hydrophobicity as a way to detect potential TM regions in the sequence, but were unable to inform regarding their orientation. This changed with the observation that positively-charged residues are more frequently found on the cell’s ‘inside’ (cytoplasmic loops), an observation widely known as the ‘positive-inside rule’ [102, 103]. This finding was implemented in the TopPred algorithm from 1992 [104, 105], where, for the first time, the software could decide whether a given region is cytoplasmic, extracellular or TM.

In 1994, the MEMSAT algorithm [106, 107] used statistical tables compiled from well-characterized membrane protein data and, by combining dynamic programming and propensity scales, produced the best overall topology. In the following years, more methods were made available to the public, based on statistical analysis of amino acid preferences and hydrophobicity, like PRED-TMR [108] and the more recent SCAMPI [109], which has been updated in 2016 [110].

The use of HMMs for the topology prediction task was initially introduced in the TMHMM [95, 111] and HMMTOP [112, 113] methods in 1998. Some years after these first HMM-based attempts, given that SPs are often falsely predicted as TM segments because of their high hydrophobicity, better-scoring methods that simultaneously predict the topology of the protein and the presence of an SP were developed, beginning with Phobius in 1994 [80]. Later developments along these lines include PolyPhobius (using evolutionary information [114]), Philius [115], MEMSAT3 [88], MEMSAT-SVM [87] and SPOCTOPUS [116].

A key improvement in the topology prediction field was the inclusion of evolutionary information in the prediction process, in the form of multiple sequence alignments, also known as profiles. The early algorithms used only a single sequence as input; however, as the sequence databases were growing with time, researchers started to exploit the availability of data. In 1993, it had been shown that profiles do improve protein secondary structure prediction [117]. The methods TMAP from 1994 [118] and PHDhtm from 1995 [119] were the first to use the evolutionary information in topology prediction. This step, as was later shown in a comparative study [120], indeed improves the accuracy and has since many years become a standard step during the development of sequence-based prediction algorithms.

PHDhtm [119] was the first topology prediction method that incorporated ANNs in the prediction process for TM proteins. By using profiles, it creates a consensus prediction for the target sequence and then finds the topology of the protein using the “positive-inside rule”. Similarly, methods that also use evolutionary information, like PRO/PRODIV-TMHMM [120] and OCTOPUS [121] were created. The latter is a combination of ANNs, that predict inside/outside and membrane/non-membrane residue preferences and an HMM which is then used to calculate the final topology.

Other machine learning methods that have been used in topology predictors are Support Vector Machines (SVMs) and Dynamic Bayesian Networks (DBNs) that are found in MEMSAT-SVM [87] and Philius [115], respectively. Finally, consensus-based approaches, like CoPreTHi [122], TOPCONS [123], MetaTM [124] and CCTOP [125], which combine the outputs from several predictors into a consensus output using dynamic programming, have been quite successful.

Prediction methods that include both models for SPs and TM segments [80, 87, 88, 114–116] are more useful for proteome-wide analyses. The updated version of the TOPCONS consensus topology prediction method, TOPCONS2 [126], can also account for the presence of an SP, thus is ideal for large scale predictions.

Numerous methods that aim at topology prediction of β-barrel TM proteins also exist. These include methods based on hydrophobicity analysis [127], statistical preferences of amino acids [128], remote homology detection [129], HMMs [130–134], SVMs combined with HMMs [135, 136], feed-forward ANNs [137, 138] and radial basis function ANNs [139]. PRED-TMBB2 [134] is, to our knowledge, the only approach available as a web-server that incorporates SP prediction in the topology prediction. This is an important feature since bacterial β-barrel proteins should have an SP that guides them through the inner membrane and towards the outer membrane of the cell. More detail on prediction of both α-helix and β-barrel TM proteins is found in a recent review [97].

## Multi-category Location Predictors

Obviously, the presence or absence of an SP or one or more TM segments is not the whole story about the subcellular location of a protein. The typical user will want to know not only whether certain sorting signals are present, but exactly where in the cell the protein goes. Several predictors have attempted to deliver this service, the first being PSORT from 1991 [29, 30]. This was an integrated expert system of several prediction methods, using both sorting signals and global properties. Some of the components were developed within the PSORT group, others were implementations of methods published elsewhere, including selected PROSITE patterns [68]. PSORT was the first publicly available system that showed this degree of integration, and it included predictions for locations that no other available methods provided at that time, e.g., nuclear and peroxisomal targeting.

All the constituent predictors provided feature values, which were then integrated to produce a final prediction. In the original version, PSORT I, the integration was done in the style of a conventional knowledge base using a collection of “if–then” rules. This makes it very difficult to adjust the rules according to information from new data sets; so in order to be able to incorporate new data on a regular basis, the newer PSORT II version used quantitative machine learning techniques, such as probabilistic decision trees and the *k* nearest neighbours classifier to integrate scores from all the features [140, 141].

### Amino Acid Composition-Based Methods

In addition to the recognition of sorting signals, prediction of protein sorting can exploit the fact that proteins of different subcellular compartments differ in global properties, reflected in the AA composition. While the signal prediction methods are probably closer to mimicking the information processing in the cell, methods based on global properties can complement imperfect signal-based methods, especially on incomplete sequences. Specifically, a composition-based method for recognizing extracellular proteins can be used without knowledge of the N-terminus, and could give correct predictions for, e.g., protein fragments or genomic sequences with erroneous assignment of start codons. One drawback is that such methods will not be able to distinguish between closely related proteins that differ in the presence or absence of a sorting signal.

As mentioned in the introduction, this approach constituted the very beginning of protein sorting prediction in the attempts by Capaldi and Vanderkooi and Barrantes [12–14] to recognize integral membrane proteins. In 1994, Nakashima and Nishikawa [142] reestablished this line of research by using simple odds-ratio statistics to discriminate between soluble intracellular and extracellular proteins on the basis of AA composition and AA-pair frequencies. Including AA pairs (separated by up to four positions) improved performance by 8% relative to AA composition alone.

In 1997, Cedano et al. [143] extended the number of possible locations to five: intracellular, extracellular, transmembrane, membrane-anchored, and nuclear; and used the so-called Mahalanobis distance to discriminate. This metric takes interactions between AAs into account (note: not interactions between positions; the input is only the 20 AA frequencies) and is therefore able to handle non-linear mappings in the 20-dimensional space defined by the AA composition. Their algorithm, named ProtLock, was for a time available as a downloadable program. This approach was refined in three subsequent papers by Chou and Elrod [144–146], who used a modified version of the Mahalanobis distance, where an extra term compensated for differences in size between the categories.

The NNPSL method by Reinhardt and Hubbard from 1998 [147] used ANNs trained on overall AA composition to predict location. They discriminated between three bacterial compartments (cytoplasmic, periplasmic, and extracellular) and four animal/fungal compartments (cytoplasmic, extracellular, mitochondrial, and nuclear). Interestingly, plant proteins were found to be very poorly predicted, and were not included in the final method. The NNPSL dataset was subsequently used by others employing different machine learning techniques, notably Yuan in 1999 using Markov chains [148] and Hua and Sun in 2001 using SVMs in their method named SubLoc [149].

One rather disturbing aspect of these early composition-based methods is their lack of proper homology reduction of the data. If the test set contains sequences that are very closely related to sequences in the training set, these proteins will also be close to each other in AA composition space, and prediction performance will be overestimated. To estimate a true generalization performance on new unrelated sequences, the dataset should be reduced or partitioned to avoid homology between training and test, and the threshold for when two proteins are too closely related should be set at a value where the problem cannot be solved by alignment alone. In the field of protein structure prediction, methods for determining the threshold and carrying out the reduction were published in the early 1990s [150, 151], and concerning SP prediction, the choice of threshold was discussed in detail in a 1996 paper [152], but in AA composition-based methods, it was apparently ignored for another decade. The NNPSL dataset was homology reduced, but only down to 90% identity [147], while Chou and Elrod only excluded proteins with the same *name* from the data set [144–146].

Better homology reduction was introduced to this subfield in 2005 and 2006 by three SVM-based methods, LocTree [153], CELLO [154] and BaCelLo [155]. They all supplement the total AA composition (and, in the case of CELLO, also AA pair composition) by AA composition in parts of the sequence. While CELLO divided the sequence into a number of subsequences of equal length, BaCelLo used a set of N- and C-terminal windows of fixed lengths, and LocTree calculated AA composition for three predicted secondary structure states separately. Both BaCelLo and LocTree searched a sequence database to create a profile and calculated AA composition in these profiles rather than the query sequence itself. The CELLO authors made a thorough examination of the relationship between alignment and machine learning predictions and reported that above a limit of 30% identity, alignment performed better than the SVM-based prediction system. Similarly, the BaCelLo authors reported that the prediction of subcellular localization in the NNPSL dataset could be carried out with a BLAST search [91], where the localization of each protein was simply predicted to be that of the closest homologue within the dataset. The performance of this simple procedure was actually better than that of the machine learning-based methods NNPSL and SubLoc and at the same level as two newer methods (LOCSVMPSI [156] and ESLpred [157]).

Why does the AA composition approach work to some degree, if it is not able to detect the sorting signals? It is no mystery that discrimination of TM versus soluble proteins is possible, since the strong hydrophobicity of the TM helices influences the AA composition; and the discrimination of inner versus outer membrane TM proteins should also be quite easy, since these are generally α-helix versus β-sheet proteins, respectively. It is more surprising that discrimination between soluble proteins of different compartments by AA composition is possible. A plausible explanation is that the protein surfaces reflect the chemical properties (acidity, ion concentrations, etc.) of their compartments. Andrade et al. [158] found that the signal in the total AA composition, which makes it possible to identify the subcellular location, is due almost entirely to surface residues.

### Homology-Based Methods

Arguably, the simplest approach to subcellular location prediction is the BLAST search described in the previous subsection: assign the subcellular location of the best hit in a database of annotated examples. This is based on the assumption that proteins tend to stay in the same compartment over the course of evolution, which seems to be the case judging from an extensive analysis of sequence conservation in relation to subcellular location [159].

Imai and Nakai in 2010 [160] showed that this approach was superior to three at the time well established predictors (CELLO 2.5 [154], MultiLoc2 [161] and WoLF PSORT [162]) if the dataset was not homology reduced, and it performed on a par with the predictors if the dataset was homology reduced to 30% identity. This result was used by the authors of the LocTree3 method in 2014 [163]: It simply outputs the location of the best BLAST hit in an annotated database if that hit has an E-value better than a certain cutoff, and reverts to its predecessor, the SVM-based LocTree2 [164], otherwise. The bacteria-specific predictor PSORTb 3.0 [165] uses a similar combination of approaches.

There are other ways to use homology information than this direct transfer of homologue location annotation. One approach is to calculate a phylogenetic profile for each protein—a specification of the pattern of occurrence of matches to that protein among a set of organisms with sequenced genomes. This was pioneered by Marcotte et al. [166]. Another approach is to search for conserved domains or motifs that are characteristic of specific locations [167].

It is also possible to use other parts of the homologue annotations than the subcellular location information. Several predictors use Gene Ontology (GO) terms [168] of the retrieved homologues as inputs for their methods, including the GOASVM and mGOASVM predictors [169, 170] and the iLoc family of predictors [171–177]. The GO terms may contain a richer source of information, but they also frequently include terms that are themselves predicted, potentially leading to a situation of circular reasoning, especially if GO-based predictors are used for assigning new GO terms. Other approaches in this direction are taken by the PA-SUB predictor [178] which looks at the occurrence of certain key words and phrases in the UniProt entries of the retrieved homologues, and the SherLoc predictor [179, 180] which does text mining of abstracts linked from the UniProt entries.

There are two advantages to using AA composition-based or homology-based methods. First, they can be used also for those compartments where the actual sorting signals are not known, or are too poorly characterized to support a proper signal-based prediction method. Second, they may work for sequences that are fragments from which the actual sorting signal may be missing or for amino acid sequences derived from genomic or metagenomic sequence where the start codon of the protein has not been correctly predicted, thus obscuring any N-terminal sorting signals. On the downside, AA composition-based or homology-based methods do not provide the same degree of insight into the information processing in the cell since they typically ignore which parts of the sequence are actually important for sorting. Another drawback is that such methods will not be able to distinguish between very closely related proteins that differ in the presence or absence of a sorting signal, and they will not be able to predict the effects of small mutations that destroy or create a sorting signal.

### Integrated Methods

As mentioned, PSORT I was in 1991 the first integrated method for protein subcellular location prediction. It was succeeded by PSORT II in 1996 [140, 141] and later by PSORTb for bacterial proteins [165, 181, 182] and WoLF PSORT for eukaryotic proteins [162]. All these methods are based on feature predictors that predict e.g. SPs or TM helices, and classification systems that integrate the output of the feature predictors. A homology component is, as mentioned, also part of PSORTb 3.0 [165].

A similar approach was taken by MultiLoc in 2006 [183] which integrated SVM-based prediction of N-terminal sorting signals with SVM-based prediction based on AA composition and a database of sorting-relevant motifs such as nuclear localization signals. The integration was done by another layer of SVMs. MultiLoc2 from 2009 [161] additionally incorporated phylogenetic profiles and GO terms of retrieved homologues.

YLoc from 2010 [184, 185] used a different technology, the Naïve Bayes classifier, to select between a very large number of simple features and integrate the selected features. Naïve Bayes is a linear method that is often outperformed by ANNs, HMMs, or SVMs. The advantage, however, is that it not only provides a prediction, but also a *reason* for the prediction in the form of a list of the features that led to the prediction in each particular case. YLoc can optionally include GO terms in the prediction.

LocTree2 from 2012 [164] is a system of SVMs arranged in a hierarchy or decision tree. Each decision is made by an SVM using a *profile kernel*, a kind of string kernel that calculates the frequencies of short motifs in a sequence profile made by PSI-BLAST [91].

Lastly, deep learning has also entered the multi-location prediction field in the form of DeepLoc from 2017 [186]. DeepLoc uses a combination of convolutional and recurrent ANNs together with a so-called attention layer which assigns a weight to every position in the sequence. In this way, the user gets an indication of which parts of each input sequence were important for the prediction. DeepLoc does not use any annotation from homologues, but still its performance was shown to be superior to seven other methods including GO-based ones like MultiLoc2 [161] and iLoc-Euk [171].

## Discussion

The first attempts to predict SPs in protein sequences were made more than 35 years ago, based on very simple statistics. Since then, the field has progressed in steps with methodological developments in the wider area of bioinformatics, such as the use of weight matrices, ANNs, HMMs, SVMs, and, more recently, deep and recurrent ANNs. In turn, these increasingly “data-hungry” approaches have been made possible by the revolution in high-throughput DNA sequencing that we have witnessed over the past couple of decades. From a historical perspective, this can stand as a nice example of how developments in computer science and wet-lab molecular biology have reinforced each other in creating the vast field of sequence-based bioinformatics that we see today.

It is difficult to estimate the full impact of SP prediction methods on biology, but it is abundantly clear that they have played an important role in proteomics research, genome annotation, identification of potential drug targets, and in a multitude of cases where the knowledge that a particular protein is secreted or membrane-anchored has been critical for understanding its function.

In the age when a rapidly growing number of genomes have been sequenced, experimentally confirmed annotations of subcellular location, molecular function, post-translational modifications etc. do not grow at nearly the same pace. Consequently, the islands of experimental annotations are increasingly far apart in the expanding sea of sequence data. This means that homology-based methods, which depend critically on the quality of the annotations they use for prediction, have an increasingly sparse basis of high quality data. It also means that machine learning methods should find a way to utilize the information inherent in unannotated data.

As our knowledge of cell architecture and compartmentalization improves, the need for new and even better methods to predict subcellular localization of proteins will remain, also given the revolution in single cell technologies. In particular, there is still room for major improvements in signal-based multi-category location methods that can sort proteins between multiple cellular locations with high reliability through modeling of the actual sorting signals, instead of relying on AA composition or homology. While in many ways mature, the field still holds interesting challenges for the bioinformatician.

## Abbreviations

AA: Amino acid
ANN: Artificial neural network
GO: Gene ontology
HMM: Hidden Markov model
SP: Signal peptide
SVM: Support vector machine
TM: Transmembrane
